# Congenital myopathy with hanging big toe due to homozygous myopalladin (MYPN) mutation

**DOI:** 10.1186/s13395-019-0199-9

**Published:** 2019-05-27

**Authors:** Luciano Merlini, Patrizia Sabatelli, Manuela Antoniel, Valeria Carinci, Fabio Niro, Giuseppe Monetti, Annalaura Torella, Teresa Giugliano, Cesare Faldini, Vincenzo Nigro

**Affiliations:** 1 Department of Biomedical and Neuromotor Sciences, University of Bologna, Bologna, Italy; 2 IRCCS-Istituto Ortopedico Rizzoli, Bologna, Italy; 3 Institute of Molecular Genetics, National Research Council of Italy, Bologna, Italy; 4 Division of Cardiology, Hospital St. Orsola, Bologna, Italy; 5 Nigrisoli Hospital, Bologna, Italy; 6 Dipartimento di Medicina di Precisione, Università della Campania “Luigi Vanvitelli”, Naples, Italy; 7 Department of Biomedical and Neuromotor Sciences, University of Bologna, Clinic of Orthopaedic and Traumatology, Istituto Ortopedico Rizzoli, Bologna, Italy; 8 Telethon Institute of Genetics and Medicine (TIGEM), Pozzuoli, Italy

**Keywords:** Myopalladin (MYPN), Z line, Hanging big toe, Cardiomyopathy, Contractures, Congenital muscular dystrophy

## Abstract

**Background:**

Myopalladin (MYPN) is a component of the sarcomere that tethers nebulin in skeletal muscle and nebulette in cardiac muscle to alpha-actinin at the Z lines. Autosomal dominant *MYPN* mutations cause hypertrophic, dilated, or restrictive cardiomyopathy. Autosomal recessive *MYPN* mutations have been reported in only six families showing a mildly progressive nemaline or cap myopathy with cardiomyopathy in some patients.

**Case presentation:**

A consanguineous family with congenital to adult-onset muscle weakness and hanging big toe was reported. Muscle biopsy showed minimal changes with internal nuclei, type 1 fiber predominance, and ultrastructural defects of Z line. Muscle CT imaging showed marked hypodensity of the sartorius bilaterally and MRI scattered abnormal high-intensity areas in the internal tongue muscle and in the posterior cervical muscles. Cardiac involvement was demonstrated by magnetic resonance imaging and late gadolinium enhancement. Whole exome sequencing analysis identified a homozygous loss of function single nucleotide deletion in the exon 11 of the *MYPN* gene in two siblings. Full-length MYPN protein was undetectable on immunoblotting, and on immunofluorescence, its localization at the Z line was missed.

**Conclusions:**

This report extends the phenotypic spectrum of recessive MYPN-related myopathies showing: (1) the two patients had hanging big toe and the oldest one developed spine and hand contractures, none of these signs observed in the previously reported patients, (2) specific ultrastructural changes consisting in Z line fragmentation, but (3) no nemaline or caps on muscle pathology.

## Background

Four patients in four families were recently described with a mild childhood-to adult onset slowly progressive nemaline myopathy with intranuclear rods and cardiac problems caused by biallelic mutations in myopalladin (*MYPN*) [1]. Homozygous truncating mutations in *MYPN* were subsequently reported in other two families, in which three patients presented with a slowly progressive cap myopathy characterized by facial, axial, hip-girdle, and distal weakness, but not cardiac alterations [2, 3].

*MYPN* is a component of the sarcomere that tethers nebulin in skeletal muscle and nebulette in cardiac muscle to alpha-actinin at the Z lines [4]. Heterozygous variants in *MYPN* have been associated with different cardiac phenotypes: dilated, hypertrophic, or restrictive cardiomyopathy [5–7], even if some reports should be reanalyzed [8].

The “hanging big toe” sign, that is highlighted in an attempt to extend the toes and is due to a selective weakness of the extensor hallucis longus, has been reported in Laing early-onset distal myopathy [9] due to mutations in the slow skeletal muscle fiber myosin heavy chain gene (*MYH7*) [10].

Here, we described a congenital myopathy with hanging big toe and cardiac problems due to a novel homozygous *MYPN* mutation resulting in myopalladin deficiency and in a peculiar ultrastructural disruption of the regular square pattern of the Z line, but no nemaline or caps on muscle pathology.

## Case presentation

### Clinical and pathological features

The proband (II.2) was the second child of healthy consanguineous parents. He was born at 36 weeks of gestation and needed supplemental oxygen to help breathing. At age 4 months, poor head control was noted. Because of delayed motor milestones he underwent a biopsy of the vastus lateralis muscle at age 9 months. He started walking after age 18 months. He was never able to run or hop. Toe-walking was noted at age 10 years. He was examined by us the first time at 14, and then at 23, 26, and 35 years of age.

On physical examination, at age 14, he was below the fifth centile for height and weight. He was able to climb stairs without aid but not to walk on heels. He showed amyotrophy of sternal portion of the pectoralis muscle and hypotrophy of brachioradialis and of the dorsalis muscles. He was unable to bury the eyelashes. There was a mild weakness (MRC 4/5) of the shoulder and hip girdles and proximal limb muscles. Muscle strength was more decreased in the axial and distal muscles with neck flexors (2), abdominals (3), finger flexors and extensors, and foot and toe extensors (4). There was pes cavus deformity and severe extensor hallucis longus weakness (0) resulting in hanging big toe. Serum creatine kinase was normal and EMG of tibialis and quadriceps was myopathic. A muscle biopsy was performed on the left quadriceps muscle (see below).

**Fig. 1:**
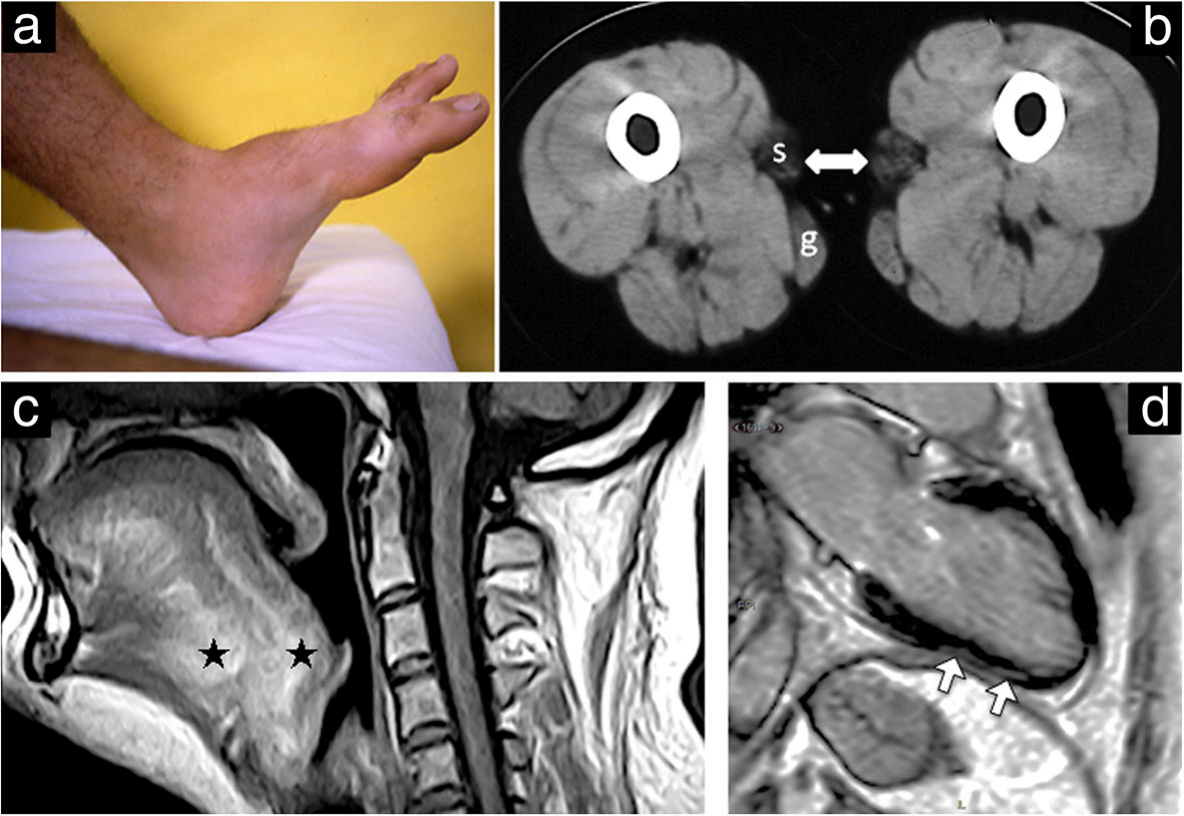
Clinical image, muscle CT, tongue MRI, and cardiac MR. Clinical image (**a**) showing the “hanging big toe sign”. Muscle CT of the mid-thigh (**b**) enlightens a selective pattern of involvement with the sartorius (s) and gracilis (g) muscles respectively severely and moderately affected, and the other muscles still well preserved. T1 weighted tongue MRI (**c**) shows scattered abnormal high-intensities areas (asterisks) in the internal tongue structure and the diffuse involvement of the posterior extensor muscles of the cervical spine. Cardiac magnetic resonance long-axis image (**d**) shows linear subepicardial late enhancement in the mid and apical segments of the inferolateral wall of the left ventricle (white arrows)

During the twenties, he experienced more difficulty with stairs. At age 26, in addition to the hanging big toe sign (Fig. 1a), he showed mild ptosis, mild weakness of orbicularis oris muscle, nasal voice, more evident atrophy of the sternal portion of the pectoralis major muscle, some limitation in flexion of the cervical and lumbar spine, and limited extension of the wrist and fingers. Knee extensors were 138 N on the right and 181 N on the left (normal > 250 N).

When examined at age 35, he had a waddling gait and needed a banister to climb stairs. Some deterioration in muscle strength was noted in the axial and distal muscles: neck flexors (1), abdominals (2), finger flexors (3), hand muscles (3+), and toe extensors (3+). Knee extensors measured 103 N on the right and 126 N on the left.

Muscle CT at age 14 (Fig. 1b) showed at mid-thigh marked hypodensity of the sartorius bilaterally and of less extent of the upper part of the gracilis. MRI at age 35 (Fig. 1c) showed scattered abnormal high-intensity areas in the internal tongue muscle and in the posterior cervical muscles.

The patient underwent periodic cardiac evaluation. No family history of cardiac disease or of sudden death was present. At 26, he was asymptomatic, the ECG was normal apart from mild voltage increase, and the echocardiogram showed biventricular normal volumes and ejection fraction (left ventricular EF 55%). At the age of 36, he repeated complete cardiac evaluation. He continued to deny any cardiac symptoms. The ECG showed first-degree atrioventricular block (PR 208 ms) and left anterior hemiblock (QRS 122 ms) with high ventricular voltages. Twenty-four-hour Holter monitoring revealed a marked sinus arrhythmia with 42 to 120 beats/min and rare ventricular ectopic beats. The echocardiogram revealed worsening of left ventricular (LV) EF, then the patient underwent cardiac magnetic resonance (CMR). The CMR showed normal LV size (LV indexed end-diastolic volume of 63 mL/m^2^), a LVEF of 44%, and a normal LV mass index of 54 g/m^2^. On late gadolinium enhancement (LGE) imaging (Fig. 1d), there was a subepicardial enhancement of the inferior and inferoseptal mid-ventricular segments attributable to interstitial changes (fibrosis). His forced vital capacity was 2530 mL (55% of predicted) at the age of 23 and 2220 mL (51%) at age 35.

On physical examination, the parents and the older brother, aged 39, had normal muscle strength in particular of the big toe extension, and no muscular atrophy or contractures. The younger sister (II.3), aged 26, reported having little strength in her hand but had never sought medical attention for this. On examination, she had muscle weakness in neck flexors (4), finger flexors and extensors (4), finger abductors (4), shoulder abductors (4), foot extensors (4), and big toe extension (4-). There was also mild weakness of eye closure and atrophy of the pectoralis major muscle on the right. Serum creatine kinase was normal and forced vital capacity was 2070 mL (93%). The ECG and color Doppler echocardiography were normal.

### Molecular and protein analysis

**Fig. 2:**
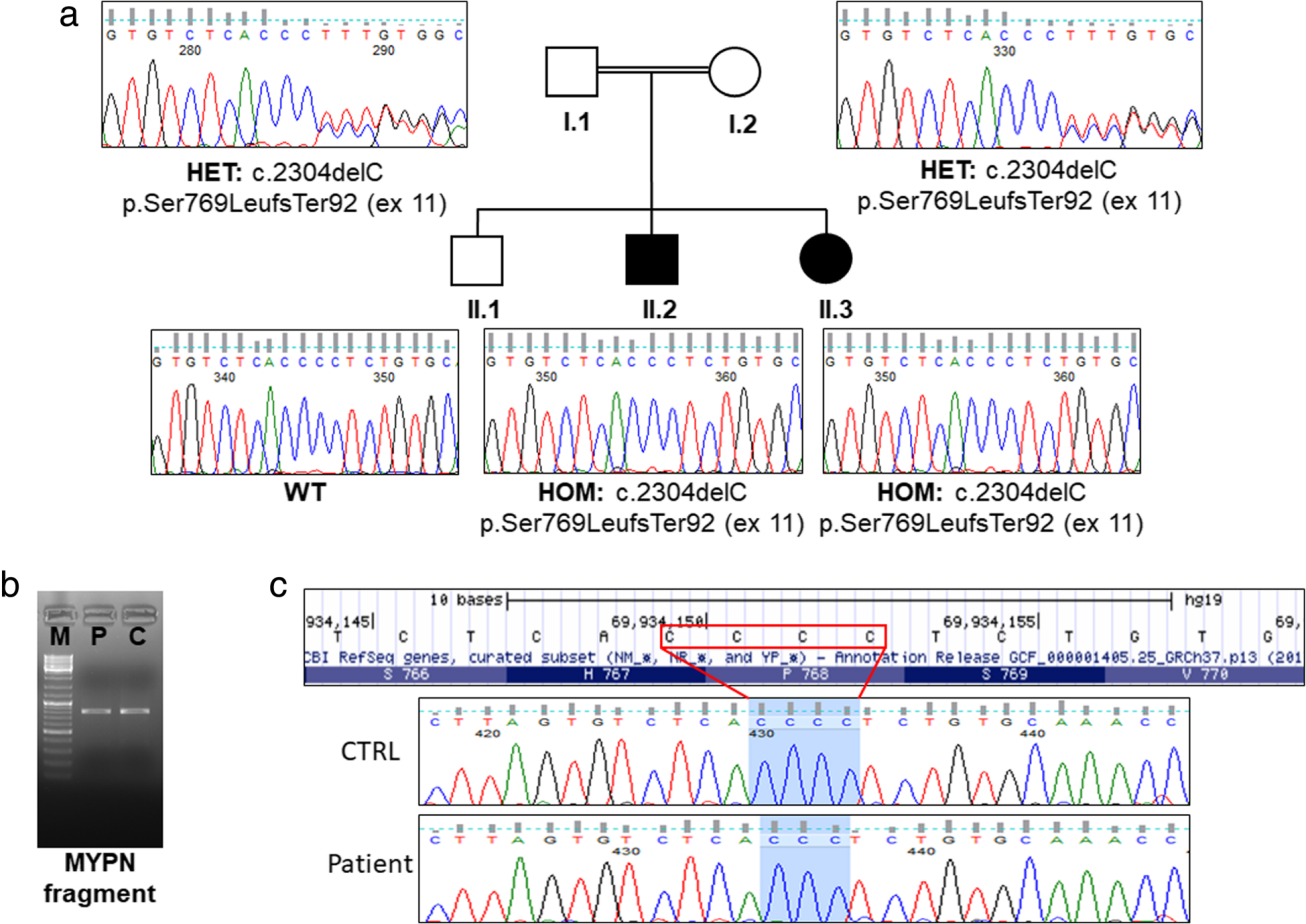
Segregation analysis and results of MYPN cDNA analysis. **a** MYPN DNA Sanger sequencing. Patients II.2 and II.3 were homozygous for p.Ser769LeufsTer92 in exon 11. Unaffected consanguineous parents were heterozygous (I.1, I.2), and unaffected brother did not show the variant (II.1). **b** RT-PCR on the *MYPN* coding sequencing using primers pairs: MYPN_c.1879F TCTTTCCAGGAGAGGTTCAACG and MYPN_c.2666R TTCCCCAAGTCTCGAATCACTG. The RT-PCR generated a fragment from exon 10 to exon 12. The patient mRNA transcript level was similar to the healthy control. **c** Sanger sequence of amplified region on the patient and control

We performed whole-exome sequencing on both affected sibling and consanguineous healthy parents. This approach showed a homozygous loss of function single nucleotide deletion: chr10:69934153, c.2304delC, and p.Ser769LeufsTer92 in the exon 11 of *MYPN* in the affected members (II.2, II.3, Fig. 2a). By Sanger sequencing, we confirmed that they have homozygous frameshift variant while the healthy parents (I.1, I.II., Fig. 2a) were heterozygous. Unaffected brother did not present the variant (II.1, Fig. 2a).

This variant was reported in ExAC (http://exac.broadinstitute.org/variant/10-69934149-AC-A) in heterozygosity only 2/121,380 alleles (frequency 0,000016), while in GnomAD (https://gnomad.broadinstitute.org/variant/10-69934149-AC-A) in heterozygosity only, with an allele frequency of 0.000007965, but never in homozygosity, like all the other loss-of-function variants in this gene. The resulting protein product is predicted to be shortened and degraded. Both heterozygous parents and their first son were healthy, confirming the recessive nature of the variant.

To understand whether the homozygous single nucleotide deletion could affect the mRNA expression, we performed RNA analysis by RT-PCR. RNA was extracted from patient and healthy control muscle biopsies. The mRNA transcript level showed no difference compared to healthy control (Fig. 2b). Primers were designed to amplify exon–exon junction region (from exon 10 to exon 12). The Sanger sequence of patient cDNA confirmed the homozygous mutation: c.2304delC at RNA level (Fig. 2c).

**Fig. 3:**
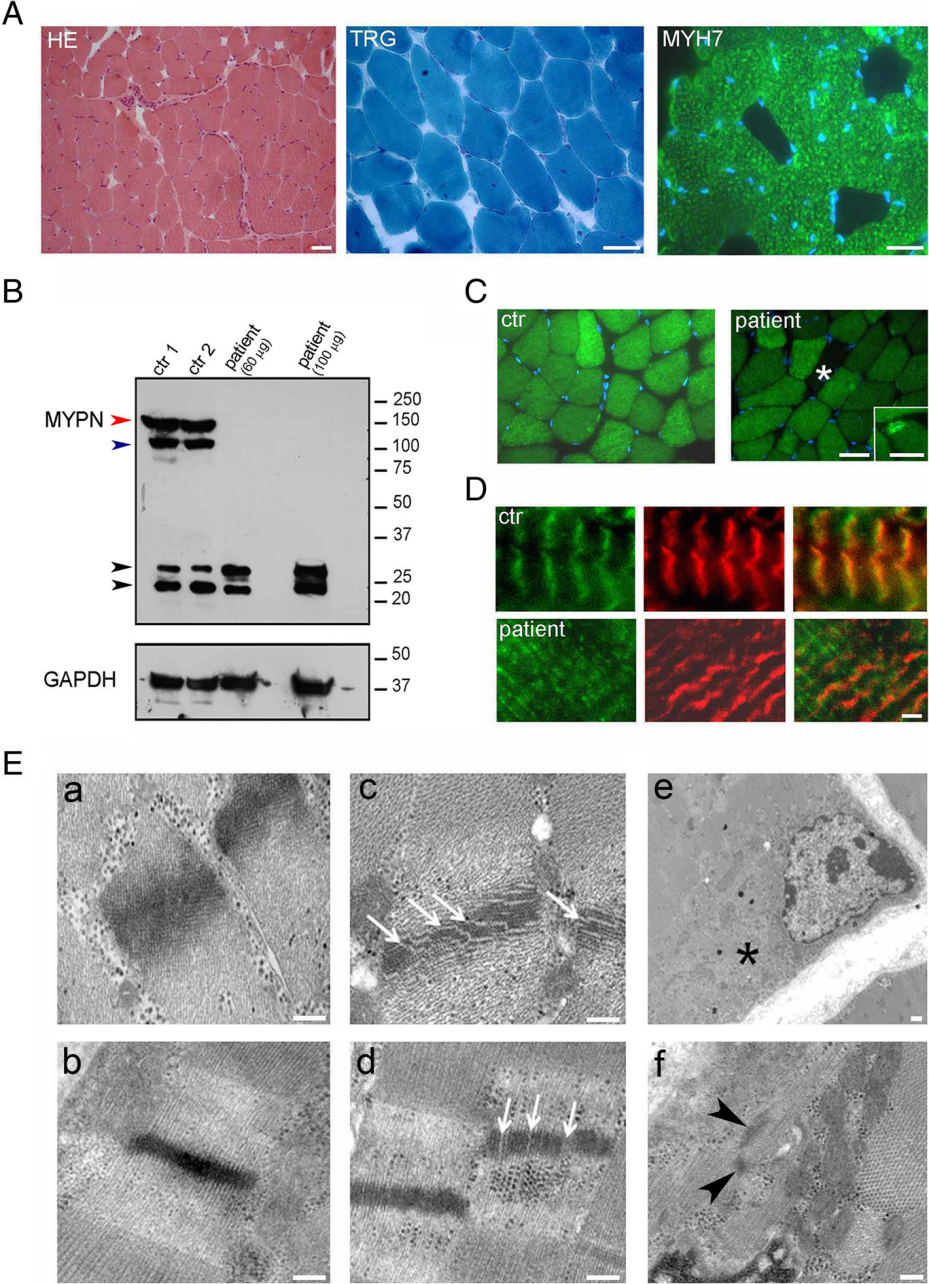
Histopathology of the patient muscle biopsy and MYPN analysis. **A** Minimal changes of the muscle architecture with hematoxylin-eosin (HE) and Gomori modified trichrome staining (TRG). A clear predominance of slow type muscle fibers is evident with anti-myosin heavy chain 7 antibody (MYH7) labeling. Scale bar, 100 μm. **B** Western blot analysis of muscle lysates from two healthy controls (ctr 1 and ctr 2) and from the patient loaded at the same protein concentration of controls (60 μg) and at higher concentration (100 μg). In control lysates, MYPN antibody identifies a clear band at the predicted molecular weight of the full-length MYPN isoform (red arrow), and a lower additional band which could correspond to the 114 kD MYPN isoform 2 (blue arrow). Additional bands (black arrows) corresponding to uncharacterized isoforms or nonspecific signal are also present. In patient, the full-length MYPN is absent, as well as the band around 100 kD, while the bands at 25 and 30 kD are similar to the controls. GAPDH was used as loading control. **C** Immunofluorescence analysis of MYPN. Note the diffuse reduction of protein, a negative fiber (asterisk), and protein accumulation in rare subsarcolemmal areas (insert) in patient muscle fibers if compared with the even distribution of control muscle. Blue staining, DAPI. Scale bar, 100 μm. **D** Double labeling of MYPN (green) and α-actinin (α-ACTN, red) in control (ctr, upper panels) and patient (lower panels) muscle sections. In patient muscle, the residual MYPN does not colocalize at Z line with α-actinin and appears diffuse among myofibrils. Scale bar, 2 μm. **E** Transmission electron microscope analysis of control (a, b) and patient (c–f). Note the interruption of the square arrangement (arrows in c) and the focal loss of actin filaments at the Z line (arrows in d) in patient muscle fibers when compared with the ordered organization in control sections (a, b). Aspects of myofilament disorganization (asterisk in e) and tiny nemaline-like structures (arrowheads in f) in subsarcolemmal areas of rare patient fibers. Scale bar, 200 nm

The morphological analysis of the quadriceps muscle biopsy of the index patient taken at age 14 showed a moderate variability of fiber size, increased number of internal nuclei, and a moderate increase of the perimysium. Succinic dehydrogenase, cytochrome c oxidase, and Gomori modified trichrome staining did not reveal significant changes, while adenosine triphosphatase at pH 10.4 (not shown) showed a clear predominance of type I fibers, as also confirmed by immunohistochemistry with anti-myosin heavy chain 7 antibody (Fig. 3a).

To assess the impact of the p.Ser769LeufsTer92 variant on myopalladin protein expression, we performed western blot analysis of control and patient muscle lysates. Myopalladin was detected with a rabbit polyclonal anti-myopalladin antibody (HPA061494 SIGMA, raised against aa 536-614 of MYPN). Muscle controls showed the full-length 145 kD myopalladin and a lower additional band which could correspond to the 114 kD MYPN isoform 2 (https://www.uniprot.org/uniprot/Q86TC9). In the patient muscle extract, both the 145 kD full-length and the 114 kD isoform were absent, even when a higher amount of protein was loaded. Two additional bands at 25 and 30 kD were present both in control and patient muscle lysates, possibly due to unspecific signal or uncharacterized shorter isoforms (Fig. 3b). The immunofluorescence analysis performed with the anti-MYPN antibody showed a moderate reduction of signal in patient muscle sections and some scattered negative fibers. Rare fibers (0.5% out of 600 fibers) displayed subsarcolemmal accumulation of MYPN staining (Fig. 3c). Similar results (not shown) were obtained with a previously characterized [4] polyclonal anti-MYPN antibody (a friendly gift from Dr. *Labeit*, Mannheim, Germany).

Double labeling with anti-MYPN and α-actinin antibodies showed that unlike normal protein, the patient MYPN residue did not co-localize with α-actinin and distributed among the myofibrils (Fig. 3d).

Transmission electron microscope analysis revealed striking alterations of the Z line in the patient biopsy. Transversal and longitudinal sections of control muscle Z line showed a regular square pattern, due to the presence in this area of cross-linking components, including myopalladin [11, 12]. Myopalladin mutation disrupts the regular square pattern of the Z line, which appeared fragmented. In addition, focal subsarcolemmal areas with accumulation of disorganized myofilaments and tiny nemaline-like structures were detected in rare patient’s myofibers (Fig. 3e).

## Discussion

Here, we described a congenital myopathy with hanging big toe and cardiac involvement due to a novel homozygous *MYPN* mutation resulting in myopalladin deficiency and in a peculiar ultrastructural stretching of Z line. This was made possible thanks to the unbiased genetic approach based on the analysis of the entire family by next-generation sequencing [13].

The index patient was floppy at birth, had delayed motor milestones, and presented in his early teens with a prevalent axial and distal four limbs weakness with hanging big toes and mild facial weakness. In the following 20 years, he had some progression of proximal and distal weakness, while remaining completely ambulant, and he showed initial signs of cardiomyopathy. His sister in the third decade of life presented muscle weakness with similar distribution but much milder.

Recessive *MYPN* mutations have been reported in seven patients from six families [1, 3] showing some common features in the clinical phenotype but also variable age of onset, muscle weakness distribution, disease severity, cardiorespiratory involvement, and muscle pathology. Our family further broadens the clinical spectrum of MYPN-related myopathies. Traditionally, diagnostic workup of cases occurred following clinical evaluation and muscle biopsy. This is changing, as genetic testing is increasingly the primary diagnostic approach, resulting in ever-expanding genotype-phenotype associations [14].

Our two affected sibs had a markedly different age of onset and severity of the disease. The presence of intrafamilial variability in patients sharing the same mutation is not uncommon, suggesting that the clinical variability may be related to the genetic background or modifier gene(s) of each patient [15]. In our patients, foot extension was weak with extensor hallucis longus weaker than tibialis anterior and extensor digitorum longus, resulting in hanging big toe sign particularly evident in the index case. This sign has been considered the hallmark of Laing distal myopathy, an early-onset autosomal dominant disease, due to mutations of slow β-myosin heavy chain (*MYH7*) gene [16]. Contractures were not observed in the previously reported cases although they also had axial [1, 3] and finger flexion weakness [3] while our patient showed limited flexion of the neck and spine and limited extension of the wrist and fingers, a type of contractures that are instead common in Bethlem myopathy [17, 18] in which the same muscles are involved.

Muscle imaging in the patients with recessive mutations in *MYPN* showed early involvement of the sartorius and gracilis [1, 3], in addition to atrophy and fatty infiltration of the axial, and hip girdle muscles [1, 3], and a peculliar fatty infiltration of the tongue in one patient [3]. Our patient with a congenital onset of weakness showed at age 14 a similar involvement of the axial and thigh muscles, but also of the pectoralis and psoas muscles, like the one we noticed in the figures of the patients described above [1]. At age 35, he also had fatty infiltrations of the tongue.

Muscle histology of patients with *MYPN* mutations has shown type 1 fiber predominance and the presence of subsarcolemmal caps [3] or nemaline bodies and intranuclear rods in muscle fibers [1, 3] associated with a dramatic reduction of myopalladin protein expression. The muscle biopsy pathology in our patient, at variance with the other reported MYPN cases, showed only minimal changes, with internal nuclei, and type 1 fiber predominance, but no caps or nemaline bodies, as revealed by conventional histoenzymatic reactions. By muscle RNA analysis, we found that mutated mRNA escaped from nonsense-mediated decay. However, the western blot analysis revealed that, like in the other cases, the myopalladin full-length isoform was absent, suggesting that a truncated protein could be quickly degraded, or a protein poorly translated from the mutant mRNA.

Additionally, in our patient, the localization of myopalladin at the Z line was missed, as shown by immunofluorescence analysis with anti-α actinin, pointing to the destabilization of Z line integrity. Interestingly, the ultrastructural analysis showed the presence of Z line alterations in several fibers, consisting in Z line fragmentation and disruption of the regular square pattern. To our knowledge, these peculiar ultrastructural alterations have never been reported in patients with *MYPN* mutations or other forms of congenital muscular dystrophies. Z line streaming and thickening or nemaline rods have been described in the muscle of patients with *MYPN* mutations [1, 3], and both alterations may be found in patients with recessive slowly progressive congenital myopathy [3]. We suggest that the Z line fragmentation detected in our patient represents an early manifestation of Z line destabilization due to loss of myopalladin and that the ultrastructural analysis may be informative in cases without specific typical histopathological features.

## Conclusions

Our findings reveal that this family had a congenital to adult-onset mildly progressive myopathy with hanging big toe, spine and hand contractures, and cardiomyopathy and no features of nemaline or cap myopathy, but with specific ultrastructural changes consisting in disruption of the regular square pattern of the Z line, extending the clinical heterogeneity of recessive *MYPN* mutations.

## Abbreviations

CT: Computed tomography
ECG: Echocardiogram
EF: Ejection fraction
EMG: Electromyography
LGE: Late gadolinium enhancement
LV: Left ventricular
MRC: Medical Research Council
MYPN: Myopalladin
MYH7: Myosin heavy chain 7
